# Neuroprotective and Anti-Neuroinflammatory Properties of Vignae Radiatae Semen in Neuronal HT22 and Microglial BV2 Cell Lines

**DOI:** 10.3390/nu14245265

**Published:** 2022-12-10

**Authors:** Yun Hee Jeong, You-Chang Oh, Tae In Kim, Jin Yeul Ma

**Affiliations:** Korean Medicine (KM)-Application Center, Korea Institute of Oriental Medicine, 70, Cheomdanro, Dong-gu, Daegu 41062, Republic of Korea

**Keywords:** Vignae Radiatae Semen, neuroprotective effects, anti-neuroinflammation, antioxidant, anti-apoptosis

## Abstract

The important factors in the pathogenesis of neurodegenerative disorders include oxidative stress and neuron-glia system inflammation. Vignae Radiatae Semen (VRS) exhibits antihypertensive, anticancer, anti-melanogenesis, hepatoprotective, and immunomodulatory properties. However, the neuroprotective effects and anti-neuroinflammatory activities of VRS ethanol extract (VRSE) remained unknown. Thus, this study aimed to investigate the neuroprotective and anti-inflammatory activities of VRSE against hydrogen peroxide (H_2_O_2_)-induced neuronal cell death in mouse hippocampal HT22 cells and lipopolysaccharide (LPS)-stimulated BV2 microglial activation, respectively. This study revealed that VRSE pretreatment had significantly prevented H_2_O_2_-induced neuronal cell death and attenuated reactive oxygen species generations in HT22 cells. Additionally, VRSE attenuated the apoptosis protein expression while increasing the anti-apoptotic protein expression. Further, VRSE showed significant inhibitory effects on LPS-induced pro-inflammatory cytokines in BV2 microglia. Moreover, VRSE pretreatment significantly activated the tropomyosin-related kinase receptor B/cAMP response element-binding protein, brain-derived neurotrophic factor and nuclear factor erythroid 2-related factor 2, and heme oxygenase-1 signaling pathways in HT22 cells exposed to H_2_O_2_ and inhibited the activation of the mitogen-activated protein kinase and nuclear factor-κB mechanism in BV2 cells stimulated with LPS. Therefore, VRSE exerts therapeutic potential against neurodegenerative diseases related to oxidative stress and pathological inflammatory responses.

## 1. Introduction

Oxidative stress and excessive inflammatory responses are closely related to the pathogenesis of the various neurodegenerative disorders of the central nervous system (CNS) [1,2]. Oxidative stress is characterized by a disequilibrium between the oxygen-derived radical generation and the antioxidant potential of organisms, thereby damaging cellular components and altering cellular function, ultimately contributing to cell death [3,4]. Reactive oxygen species (ROS) at low levels are important in neuronal signaling and physiology. However, ROS overproduction will damage proteins, DNA, and lipid membranes, leading to cellular destruction and cell death [5,6]. Microglia are major resident immune cells that play an important role in mediating inflammatory responses in the CNS. Sustained excessive activation of microglia contributes to neuronal damage and inflammatory mediator releases, such as tumor necrosis factor (TNF)-α, interleukin (IL)-6, and IL-1β, in response to lipopolysaccharide (LPS) stimulation. Overproduction of these mediators may contribute to neurodegenerative disorder progression [7,8]. Additionally, ROS production increases by an excessive inflammatory response, and oxidative stress triggers the microglia activation. Therefore, oxidative stress and excessive inflammatory response may be the major factors that cause or exacerbate neurodegenerative diseases.

Reducing the risk of oxidative stress caused by ROS overproduction and the excessive inflammatory response is a key target for treating or preventing neurodegenerative diseases. Brain-derived neurotrophic factor (BDNF) is a neurotrophin within the central and peripheral nervous systems, is concerned with neuronal cell preservation in in vivo systems, and is known to play an important role [9]. BDNF elicits its physiological function through binding to its receptor, tropomyosin-related kinase receptor B (TrkB), which initiates multiple signaling cascades [10,11,12]. Additionally, BDNF/TrkB signaling activation contributes to the phosphorylation and activation of transcription factor cAMP response element-binding protein (CREB) [13]. Another mechanism that performs neuroprotective action is correlated with the nuclear factor erythroid 2-related factor 2 (Nrf-2) signaling pathway. The Nrf-2, as a transcription factor, regulates the expression of many antioxidant enzymes, including NAD(P)H quinone oxidoreductase 1 (NQO-1), glutamate-cysteine ligase catalytic subunit (GCLC), and heme oxygenase (HO)-1 [14,15,16]. Many studies revealed that Nrf-2-mediated pathways can scavenge free radicals and efficiently protect neuronal cells from oxidative injury [17,18]. Therefore, it can be used as a strategy for pharmacological agent development that targets TrkB/CREB/BDNF pathways and the Nrf-2/antioxidant enzyme for neurodegenerative disease treatment and prevention.

Neuroinflammation is an inflammatory process of the neural cells and is triggered by various biological stimuli, such as glial reactions and oxidative stress [19]. Nuclear factor (NF)-κB and mitogen-activated protein kinases (MAPK) are known as key inflammatory signaling pathways during inflammatory process progression. The major subunits of the NF-κB heterodimer include p65 and p50. Under stimulation, NF-κB translocates into nuclei to promote several inflammatory mediator expressions, including TNF-α, IL-6, and IL-1β [20,21]. The family members of MAPKs mainly consist of the extracellular signal-regulated kinase (ERK), p38, and c-Jun NH_2_-terminal kinase (JNK) [22], which are related to the regulation of inflammatory mediator production in stimulated microglia [23]. Therefore, targeting NF-κB and MAPK pathways might be an attractive therapeutic approach for neuroinflammatory disease prevention and treatment.

*Vigna radiatus* is a leguminous species that is mainly grown in Asia, and its seeds, Vignae Radiatae Semen (VRS), are used for food or medicinal purposes. VRS is one of the most important edible legumes and contains balanced nutrients, including protein, dietary fiber, minerals, vitamins, and significant lots of bioactive compounds [24]. VRS has been traditionally used as a medicinal herb for its detoxification activities, mentality recuperation, heat stroke alleviation, and gastrointestinal upset regulation. Previous studies reported that VRS is beneficial because of its hypoglycemic and hypolipidemic effects, as well as antihypertensive, anticancer, anti-melanogenesis, hepatoprotective, and immunomodulatory properties beyond meeting basic nutrient requirements [25,26,27,28,29,30]. However, the physiological activity and molecular mechanisms of HT22 hippocampal neuron-mediated neuroprotective and microglia-mediated anti-neuroinflammatory effects generated by VRS ethanol extract (VRSE) remained unknown. Hence, the current study aimed to determine the neuroprotective effects and anti-neuroinflammatory activities of VRSE in H_2_O_2_-exposed HT22 hippocampal cells and LPS-stimulated BV2 microglial cells. Furthermore, the chemical constituents of VRSE were investigated using high-performance liquid chromatography (HPLC) analysis.

## 2. Materials and Methods

### 2.1. Materials and Reagents

Dulbecco’s modified Eagle’s medium (DMEM), antibiotics, and fetal bovine serum (FBS) were purchased from Hyclone (Logan, UT, USA). All cell culture dishes and plates were obtained from SPL life sciences (Pocheon, Republic of Korea). LPS, bovine serum albumin (BSA), and dimethyl sulfoxide were purchased from Sigma–Aldrich (St. Louis, MO, USA). The Cell counting kit (CCK) was purchased from Dojindo Molecular Technologies (Kumamoto, Japan). The 2′,7′-dichlorofluorescein diacetate (H_2_DCFDA) was obtained from Invitrogen (Carlsbad, CA, USA). Various primary antibodies and horseradish peroxidase-conjugated secondary antibodies were obtained from Cell Signaling Technology, Inc. (Boston, MA, USA), Santa Cruz Biotechnology (Santa Cruz, CA, USA), and Thermo Fisher (Waltham, MA, USA). The polyvinylidene difluoride (PVDF) membrane was acquired from Millipore (Bedford, MA, USA). Enzyme-linked immunosorbent assay (ELISA) antibody sets were obtained from eBioscience (San Diego, CA, USA). Vitexin and isovitexin were purchased from Sigma–Aldrich. HPLC grade acetonitrile was obtained from Merck (KGaA, Darmstadt, Germany) and formic acid was purchased from Sigma–Aldrich. Tertiary distilled water was prepared by Puris-Evo-UP Water System with Evo-up Dio VFT and Evo-ROP Dico20 (Mirae ST Co., Ltd., Anyang, Republic of Korea).

### 2.2. Preparation of VRSE

VRS was purchased as a dried herb from Yeongcheonhyundai Herbal Market (Yeongcheon, Korea) and authenticated by Prof. KiHwan Bae of the College of Pharmacy, Chungnam National University (Daejeon, Republic of Korea). All voucher specimens were deposited in an herbal bank at the Korean Medicine-Application Center, Korea Institute of Oriental Medicine (voucher number: E26). The dried herb (30.0 g) was extracted in 70% ethanol (390 mL) at 40 °C in a shaking incubator (100 rpm) for 24 h. The extract solution was filtered through a 150 mm filter paper (Whatman, Piscataway, NJ, USA) and concentrated using a rotary vacuum evaporator (Buchi, Tokyo, Japan). Samples were then freeze-dried and kept in desiccators at −20 °C before use. The sample yield was 7.4693%, and 2.2408 g of extract was obtained.

### 2.3. Cell Culture

The murine hippocampal HT22 cell and microglial cell line BV2 were purchased from the American Type Culture Collection (Manassas, VA, USA) and grown in a DMEM medium that contains 10% of FBS and 1% of antibiotics. Each cell was maintained in a humidified incubator (37 °C, 95% of air, and 5% of CO_2_) during cultivation. The culture medium was renewed every 2 days, and cells were grown to 80–85% confluence for the experiments.

### 2.4. Cell Viability

HT22 cells (5 × 10^3^ cells/well) were seeded and cultured into a 96-well plate for 24 h. BV2 cells were seeded into 96-well plates at a density of 5 × 10^4^ cells/well and cultured for 18 h. After pre-incubation, VRSE or H_2_O_2_ was added to the cells, which were incubated for 24 h at 37 °C with 5% of CO_2_. Each well was added with 10 μL of CCK solution and incubated for 1 h. The absorbance of each well at 450 nm was measured using a microplate reader (SpectraMax i3, Molecular Devices, San Jose, CA, USA). Morphological changes of HT22 cells were analyzed by a holotomography (HT-X1, Tomocube, Daejeon, Republic of Korea).

### 2.5. Intracellular ROS Determination

The measurement of intracellular ROS levels was examined using H_2_DCFDA following our previous report [31]. The cells were washed twice in phosphate-buffered saline (PBS) and were then stained with 10 μM of H_2_DCFDA for 30 min at 37 °C in the dark after VRSE and H_2_O_2_ treatment. The stained cells were washed twice with PBS. A fluorescence microplate reader (SpectraMax i3, Molecular Devices, San Jose, CA, USA) at an excitation wavelength of 488 nm and emission wavelength of 525 nm was used to analyze dihydroethidium fluorescence. A fluorescence microscope (Eclipse Ti, Nikon, Tokyo, Japan) was used to obtain representative fluorescence images.

### 2.6. Western Blotting

Cell proteins were extracted using radioimmunoprecipitation assay lysis buffer (Millipore) by adding protease and phosphatase inhibitor cocktail (Roche, Basel, Switzerland). Protein samples were separated via electrophoresis in a sodium dodecyl sulfate-polyacrylamide gel and transferred onto a PVDF membrane. The membranes were blocked with 3% BSA at room temperature (RT) for 1 h and were incubated with their respective primary antibodies overnight at 4 °C. Primary antibodies were discarded, and the blots were washed with tris-buffered saline with 0.1% of Tween 20 four times. Membranes were incubated with horseradish-labeled species-specific secondary antibodies at RT for 1 h. ChemiDoc^TM^ Touch Imaging System (Bio-Rad, Hercules, CA, USA) was used to quantify the relative protein expression intensity. The band intensity was analyzed using Image J software (version 1.53k) with control value normalization. Table 1 shows the information regarding various primary and secondary antibodies.

### 2.7. Preparation of Cytosolic and Nuclear Fractions

Nuclear Extraction Kit (Thermo Fisher) was used to obtain the cytosolic and nuclear fractions. Each extracted fraction was lysed according to the protocol by the manufacturer’s instructions. The fractions were stored at −80 °C before use.

### 2.8. Cytokine Determination

The cytokine level in the culture medium was determined using an ELISA antibody set according to the manufacturer’s instructions. For ELISA, BV2 cells were seeded into a 24-well plate (2.5 × 10^5^ cells/well) and incubated overnight. The cells were pretreated with various VRSE concentrations for 1 h and further challenged with LPS for an additional 24 h at 37 °C with 5% of CO_2_ [32]. After incubation, the TNF-α and IL-6 cytokine levels were measured using the collected supernatant of the medium as previously reported [32].

### 2.9. VRSE and Standard Solution Preparation

Standard component purity was confirmed that all were >95%. Each constituent (vitexin and isovitexin) stock solution was prepared with HPLC grade methanol at the concentration of 1000 ppm (1 mg/mL) after dissolving the solution through a 0.2-μm PETE filter. The filtrate was diluted with methanol at the concentration for the prepared standard curve for each compound. VRSE at 100 mg was accurately weighed and dissolved in 1 mL of methanol:water (50:50, *v*/*v*) solvent. VRSE solution was filtered through a 0.2-μm PETE filter before HPLC injection.

### 2.10. HPLC Conditions

The VRSE was analyzed using the Dionex UltiMate 3000 HPLC system (Dionex Corp., Sunnyvale, CA, USA) consisting of a binary pump, an autosampler, a column oven, and a diode array UV/VIS detector. System control and data analysis were processed with Dionex Chromeleon. All analysis was conducted under the following conditions: Kinetex C18 column (100 × 4.60 mm, 2.6 μm, Phenomenex, Seoul, Republic of Korea), and the column oven temperature was kept at 40 °C, mobile phase consisted of 0.1% of formic acid (*v*/*v*) in water (A) and acetonitrile (B) gradient elution system to improve the chromatographic separation capacity. The gradient system was programmed as 13% B for 0−25 min and 13−100% B for 25−26 min with a flow rate of 0.6 mL/min. The total run time was 40 min, and the injection volume was 5 μL. The detection wavelength was 280 nm and all samples were injected triplicated.

### 2.11. Statistical Analysis

The data are presented as the mean ± standard error of the mean for three independent experiments. Statistical analysis of the experimental results was performed using the one-way analysis variance followed by Dunnett’s test, after comparing the H_2_O_2_ or LPS sample and each treated sample. A *p*-value of <0.05 represented a statistical significance. GraphPad Prism version 5.02 (GraphPad Software, Inc., 188 San Diego, CA, USA) was used for statistical analyses and mapping.

## 3. Results

### 3.1. Effects of VRSE on H_2_O_2_-Induced Neurotoxicity in HT22 Cells

**Figure 1:**
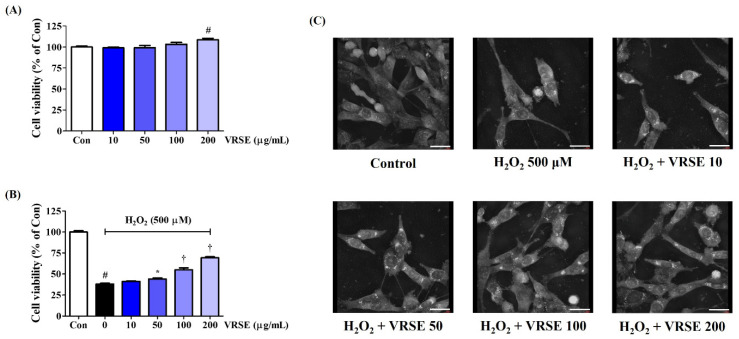
Effects of Vignae Radiatae Semen ethanol extract (VRSE) on hydrogen peroxide (H_2_O_2_)-induced cytotoxicity in HT22 cells. (**A**) HT22 cells were incubated with VRSE concentrations of 10, 50, 100, or 200 μg/mL. (**B**,**C**) After VRSE pretreatment concentrations of 10, 50, 100, or 200 μg/mL, HT22 cells were stimulated with 500 μM of H_2_O_2_. (**C**) Images represent the three independent experiments at a magnification of 400×. Scale bar = 30 μm. Control cells were incubated with the vehicle alone. Data are presented as mean ± standard error of the mean of three independent experiments. Con, control. Statistical significance was defined as # *p* < 0.05 (vs. control), * *p* < 0.05, and † *p* < 0.001 (vs. H_2_O_2_).

We assessed the effect of VRSE on the viability of HT22 mouse hippocampal neurons using the CCK assay to investigate the neuroprotective effects. VRSE treatment of up to 200 μg/mL alone for 24 h did not show any cytotoxicity on HT22 cells, as shown in Figure 1A. Thus, VRSE concentrations of <200 μg/mL were then investigated for neuroprotection activities. First, an additional CCK assay was performed to verify the possible protective effects of VRSE against the H_2_O_2_-induced neurotoxicity in HT22 cells. Our data revealed that H_2_O_2_ treatment alone at 500 μM decreased cell viability by approximately 70%. In contrast, VRSE treatment improved the cell viability of H_2_O_2_-exposed HT22 cells. At 100 and 200 μg/mL concentrations, the cell viability increased with statistical significance (Figure 1B). Additionally, H_2_O_2_-induced nuclear condensation and cell contraction were found by observing the cell morphology with a holotomography. In contrast, VRSE pretreatment had a concentration-dependent effect on maintaining cellular structure and inhibiting morphological changes (Figure 1C). Therefore, VRSE significantly mitigates neurotoxicity in H_2_O_2_-exposed HT22 cells.

### 3.2. VRSE Reduced Intracellular ROS Generation by H_2_O_2_

**Figure 2:**
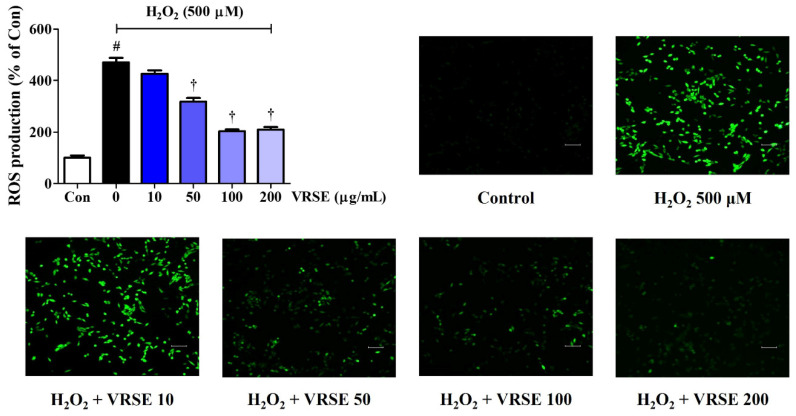
Effects of Vignae Radiatae Semen ethanol extract (VRSE) against hydrogen peroxide (H_2_O_2_)-induced intracellular reactive oxygen species (ROS) production in HT22 cells. Cells were pretreated with VRSE concentrations of 10, 50, 100, or 200 μg/mL and then with 500 μM of H_2_O_2_. H_2_DCFDA (20 μM) is an oxidation-sensitive fluorescence dye used to assess ROS levels. The ROS expression was determined using a fluorescence microscope and a fluorescence microplate reader. Scale bar = 200 μm. Control cells were incubated with the vehicle alone. All experiments were repeated at least three times, and similar results were obtained. Data are presented as mean ± standard error of the mean. Con, control. Statistical significance was defined as # *p* < 0.05 (vs. control) and † *p* < 0.001 (vs. H_2_O_2_).

We also explored the effects of several VRSE concentrations (10, 50, 100, and 200 μg/mL) on ROS accumulation in HT22 cells using an H_2_DCFDA fluorescence assay. Our data revealed that H_2_O_2_ treatment alone significantly increased intracellular ROS levels, whereas VRSE pretreatment concentration dependently reduced ROS generation (Figure 2). The fluorescent images in Figure 2 also show an effect similar to that mentioned above, suggesting that VRSE could inhibit excessive ROS production induced by H_2_O_2._

### 3.3. VRSE Suppress H_2_O_2_-Induced Apoptosis in HT22 Cells

**Figure 3:**
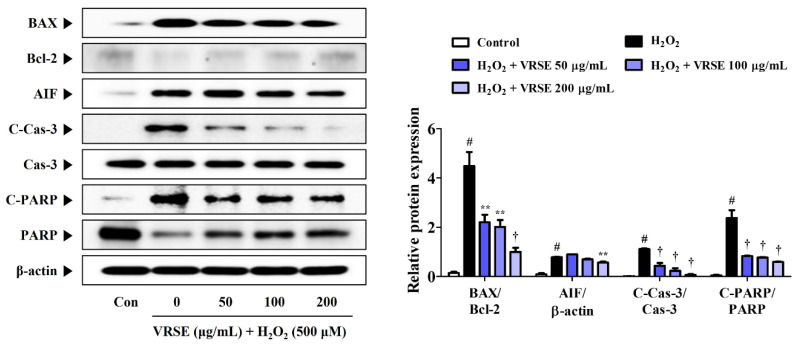
Effects of Vignae Radiatae Semen ethanol extract (VRSE) against hydrogen peroxide (H_2_O_2_)-induced apoptosis in HT22 cells. Cells were pretreated with VRSE concentrations of 50, 100, or 200 μg/mL and then exposed to 500 μM of H_2_O_2_. The expression levels of Bcl-2-associated X, B-cell lymphoma 2, apoptosis-inducing factor, caspase-3, and poly (ADP-ribose) polymerase were determined via Western blot analysis. Control cells were incubated with the vehicle alone. Blot images represent three independent experiments. Data are presented as mean ± standard error of the mean. BAX, Bcl-2-associated X; Bcl-2, B-cell lymphoma 2; AIF, apoptosis-inducing factor; Cas, caspase; PARP, poly (ADP-ribose) polymerase; Con, control. Statistical significance was defined as # *p* < 0.05 (vs. control), ** *p* < 0.01, and † *p* < 0.001 (vs. H_2_O_2_).

The expression of apoptosis-related proteins was determined using Western blot analysis to measure the prevention effects of VRSE on H_2_O_2_-induced apoptotic neurotoxicity in HT22 cells. H_2_O_2_ treatment increased the apoptotic marker expression in HT22 cells, as shown in Figure 3. In contrast, VRSE pretreatment notably diminished the apoptosis-related protein level, such as Bcl-2-associated X (BAX), apoptosis-inducing factor (AIF), cleaved-caspases-3, and cleaved-Poly (ADP-ribose) polymerase (PARP). Moreover, it enhanced the anti-apoptotic protein expression, such as B-cell lymphoma 2 (Bcl-2) and PARP compared with H_2_O_2_ alone-treated cells (Figure 3). These results revealed that VRSE pretreatment exerts a neuroprotective activity via H_2_O_2_-induced apoptotic cell death inhibition.

### 3.4. Effects of VRSE on the Production of Inflammatory Cytokines in LPS-Stimulated BV2 Cells

**Figure 4:**
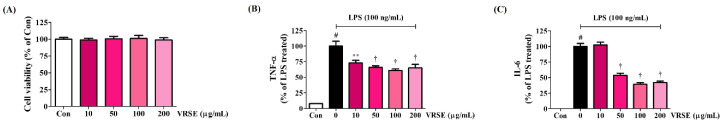
Effects of Vignae Radiatae Semen ethanol extract (VRSE) on (**A**) microglia viability and (**B**,**C**) inflammatory cytokine secretion in BV2 cells. (**A**) BV2 cells were incubated with VRSE concentrations of 10, 50, 100, or 200 μg/mL. (**B**,**C**) After VRSE pretreatment concentrations of 10, 50, 100, or 200 μg/mL, BV2 cells were stimulated with 100 ng/mL of lipopolysaccharide. Control cells were incubated with the vehicle alone. Data are presented as mean ± standard error of the mean of three independent experiments. Con, control; LPS, lipopolysaccharide; TNF, tumor necrosis factor; IL, interleukin. Statistical significance was defined as # *p* < 0.05 (vs. control), ** *p* < 0.01, and † *p* < 0.001 (vs. LPS).

First, we investigated the effect of VRSE treatment on cell viability in BV2 microglia to explore the concentration at which potential cytotoxicity was eliminated. Hence, VRSE did not affect cell viability up to a concentration of 200 μg/mL, as in HT22 cells (Figure 4A). We measured the production of inflammatory cytokines, including TNF-α and IL-6, in mouse microglia BV2 cells to identify the anti-neuroinflammatory effects of VRSE. The secretion of cytokines, which was greatly increased when LPS alone was treated, was significantly decreased in a concentration-dependent manner by VRSE pretreatment, as shown in Figure 4B,C.

### 3.5. VRSE Ameliorate the Transcriptional Activity of NF-κB and Phosphorylation of MAPK in LPS-Stimulated BV2 Microglia

**Figure 5:**
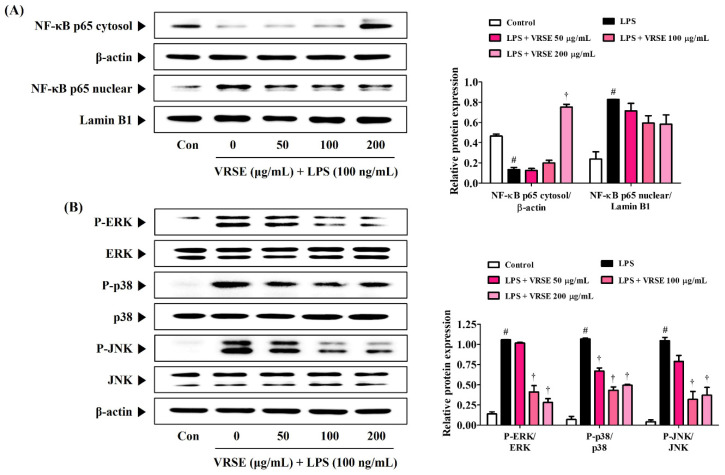
Effects of Vignae Radiatae Semen ethanol extract (VRSE) on (**A**) the nuclear translocation of nuclear factor-κB p65 and (**B**) phosphorylation of mitogen-activated protein kinases. After VRSE pretreatment concentrations of 50, 100, or 200 μg/mL, BV2 cells were stimulated with 100 ng/mL of lipopolysaccharide. Control cells were incubated with the vehicle alone. Blot images represent three independent experiments. Data are presented as mean ± standard error of the mean. NF, nuclear factor; Con, control; LPS, lipopolysaccharide; ERK, extracellular signal-regulated kinase; JNK, c-Jun NH_2_-terminal kinase. Statistical significance was defined as # *p* < 0.05 (vs. control) and † *p* < 0.001 (vs. LPS).

The transcription factor, NF-κB, is an essential regulator that is closely related to inflammatory responses. Therefore, we determined the inhibition effect of VRSE in LPS-induced p65 translocation into the nucleus via Western blot analysis. Figure 5A shows that VRSE effectively blocks LPS-induced translocation of NF-κB subunit p65 to the nucleus in a concentration-dependent manner. Additionally, MAPK, ERK, p38, and JNK are important immune response regulators and have a crucial role in regulating NF-κB activation. Thus, we assessed the effects of VRSE on the phosphorylation of MAPK. Our data revealed that VRSE pretreatment statistically significantly diminished the phosphorylation of ERK, p38, and JNK (Figure 5B).

### 3.6. Effects of VRSE on Nrf-2-Mediated Antioxidant Enzyme Expression in H_2_O_2_-Exposed HT22 Hippocampal Cells

**Figure 6:**
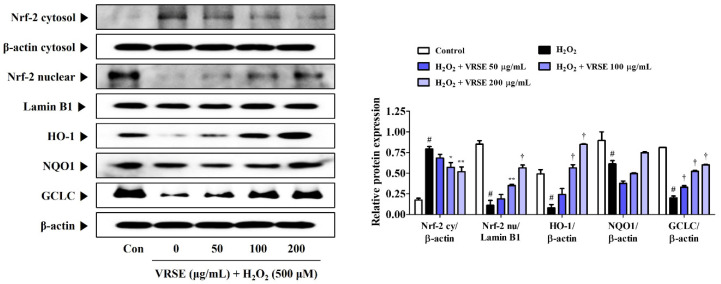
Effects of Vignae Radiatae Semen ethanol extract (VRSE) on the activation of nuclear factor erythroid 2-related factor 2 (Nrf-2) and antioxidant enzymes in hydrogen peroxide (H_2_O_2_)-exposed HT22 cells. Cells were incubated with 500 μM of H_2_O_2_ with or without VRSE. The expression levels of Nrf-2, heme oxygenase-1, NAD(P)H quinone oxidoreductase 1, and glutamate-cysteine ligase catalytic subunit were determined via Western blot analysis. Control cells were incubated with the vehicle alone. Blot images were representative of the three independent experiments and data are presented as mean ± standard error of the mean. HO, heme oxygenase; NQO1, NAD(P)H quinone oxidoreductase 1; GCLC, glutamate-cysteine ligase catalytic subunit; Con, control. Statistical significance was defined as # *p* < 0.05 (vs. control), * *p* < 0.05, ** *p* < 0.01, and † *p* < 0.001 (vs. H_2_O_2_).

The protective action of antioxidant enzymes by Nrf-2 activation suppresses oxidative stress in several models of neurodegenerative diseases, thereby exhibiting neuroprotective effects [17,18]. Therefore, we examined the induction effect of VRSE pretreatment in antioxidant enzyme expression, including HO-1, NQO-1, and GCLC, through Nrf-2 translocation to the HT22 cell nucleus. Figure 6 shows that VRSE completely increased the nuclear Nrf-2 translocation in a concentration-dependent manner. Furthermore, VRSE pretreatment strongly increased the antioxidant enzyme expression, including HO-1, NQO-1, and GCLC, in H_2_O_2_-exposed HT22 cells (Figure 6). Therefore, the antioxidant effects of VRSE may be due to Nrf-2-mediated antioxidant enzyme activation.

### 3.7. VRSE Enhances Mature BDNF Expression via TrkB/Akt/CREB Pathway Activation

**Figure 7:**
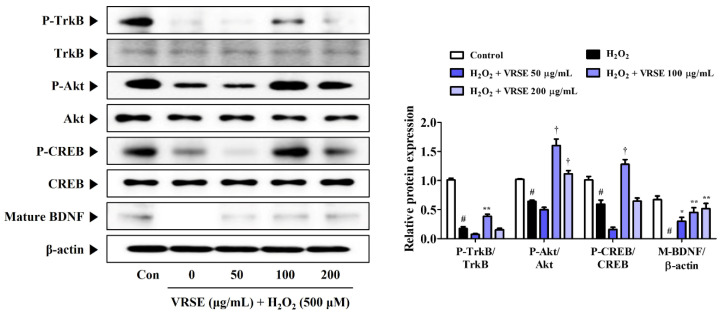
Effects of Vignae Radiatae Semen ethanol extract (VRSE) on the phosphorylation of tyrosine receptor kinase B (TrkB), protein kinase B (Akt), and cAMP response element-binding protein (CREB), and the expression of mature brain-derived neurotrophic factor (BDNF) in hydrogen peroxide (H_2_O_2_)-exposed HT22 cells. Cells were pretreated with VRSE at concentrations of 50, 100, or 200 μg/mL and then exposed to H_2_O_2_ (500 μM). The expression levels of TrkB, Akt, CREB, and BDNF were determined via Western blot analysis. Control cells were incubated with the vehicle alone. Blot images are representative of the three independent experiments and data are expressed as mean ± standard error of the mean. Con, control. Statistical significance was defined as # *p* < 0.05 (vs. control), * *p* < 0.05, ** *p* < 0.01, and † *p* < 0.001 (vs. H_2_O_2_).

TrkB/Akt/CREB/BDNF pathway activation is involved in neuronal growth and survival. Hence, we determined the effects of VRSE on BDNF formation and change of TrkB/Akt/CREB pathway activation. Figure 7 shows that VRSE effectively improved the BDNF expression, as well as P-TrkB, P-Akt, and P-CREB, only at a 100 μg/mL concentration, compared to cells treated with H_2_O_2_ alone. Conversely, VRSE pretreatment at low and 200 mg/mL concentrations showed little or no effect. Therefore, VRSE had a significant effect on TrkB/Akt/CREB/BDNF activation to some extent but was not concentration-dependent. Therefore, the neuroprotective effects of VRSE are only partially related to TrkB/Akt/CREB/BDNF signaling pathway activation.

### 3.8. Identification and Quantitative Analysis of the Chemical Constituents of VRSE

**Figure 8:**
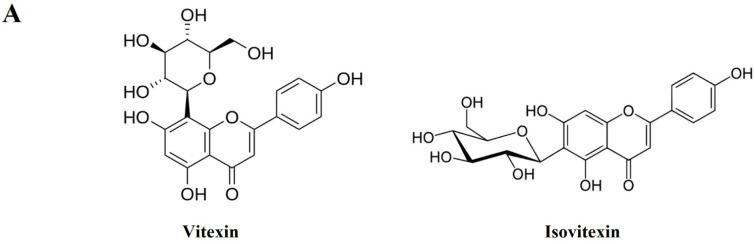

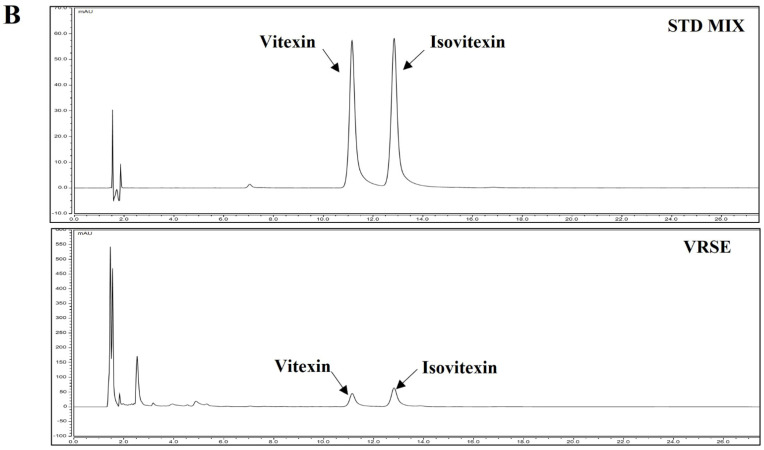

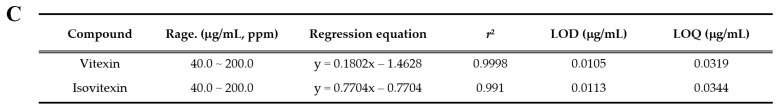
HPLC chromatogram of Vignae Radiatae Semen ethanol extract (VRSE). (**A**) Chemical structure of vitexin and isovitexin. (**B**) HPLC chromatogram of standard solution and VRSE. (**C**) Regression equations, the limit of detection, and the limit of quantitation of standard compound. STD, standard; LOD, the limit of detection; LOQ, the limit of quantitation.

HPLC analysis was performed to determine the compounds contained in VRSE and their quantification. Two compounds of vitexin (tR 11.140 min) and isovitexin (tR 12.817 min) were detected under the established HPLC condition (Figure 8). The calibration curves of the two marker compounds were y = 0.1808x−1.4628 and y = 0.1672x−0.7704, with determination coefficients of 0.9998 and 0.9991 at injected concentration ranges of 40.0–200.0 μg/mL, respectively. These results showed the good linearity of the calibration curve of two marker compounds at the tested concentration range. The mean area of VRSE was calculated for each compound calibration curve equation. The contents of vitexin and isovitexin were 1243 μg/g and 1635 μg/g, respectively. Thus, we confirmed the contents of two marker compounds in the VRSE using the developed HPLC-UV analysis.

## 4. Discussion

Oxidative stress and excessive inflammatory responses pose the CNS to susceptible damage and various neurodegenerative disorders, such as Alzheimer’s and Parkinson’s diseases [1,2]. Thus, an agent with both antioxidative and anti-inflammatory effects was proposed as a promising strategy for several neurodegenerative disease prevention and treatment [33]. VRS, the most important edible legume crop, has long been used as food and traditional medicine in East Asia. VRS has been traditionally used as a medicinal herb for its detoxification activities, mentality recuperation, heat stroke alleviation, and gastrointestinal upset regulation. However, the neuroprotective and anti-neuroinflammatory effects and detailed molecular mechanisms of VRS remained unknown. Therefore, the current study explored the therapeutic potential and underlying mechanism of VRSE in H_2_O_2_-exposed HT22 hippocampal cells and LPS-stimulated BV2 microglial cells.

This study revealed that VRSE pretreatment protected HT22 cells from H_2_O_2_-induced cytotoxicity, as evidenced by high cell viability (Figure 1). Additionally, VRSE significantly reduced the intracellular ROS level caused by H_2_O_2_ treatment (Figure 2). Moreover, H_2_O_2_-induced oxidative stress resulted in cell death as determined using Western blot analysis. Our results indicate that VRSE pretreatment remarkably inhibited the mitochondrial apoptotic proteins, including BAX, AIF, cleaved-caspase-3, and cleaved-PARP, as well as significantly increased the anti-apoptotic protein expression, including Bcl-2 and PARP (Figure 3). These results suggested that VRSE protected neuronal cell death by suppressing the excessive ROS accumulation through H_2_O_2_ treatment. One possible mechanism concerning the neuroprotective action of VRSE may be correlated with TrkB/CREB/BDNF pathway. BDNF is a member of the neurotrophin family of growth factors that influences cell proliferation, differentiation, neuronal growth, and synaptic plasticity by binding to the Trk receptor [10,11,12]. Thus, BDNF is an important factor for oxidative stress-treated neuronal cell survival. The TrkB activation by BDNF in the signaling pathway contributes to the phosphorylation and activation of CREB in neuronal cells [13]. Our present data revealed that VRSE not only enhanced the BDNF level but also elevated the P-TrkB and P-CREB activation (Figure 7). Consequently, these results indicated that VRSE induces BDNF expression, and then the released BDNF activates TrkB/CREB pathway in HT22 cells. Another possible mechanism for the neuroprotection of VRSE may be related to antioxidant enzyme regulation. Nrf-2 is a transcription factor that initiates an endogenous antioxidant response element and activates several antioxidant enzyme productions, including NQO1, GCLC, and HO-1 [14,15,16,34]. Under oxidative stress stimulation, Nrf-2 dissociates with KEAP1, and then Nrf-2 is combined into the ARE region on DNA after it translocates into the nucleus and activates the antioxidant enzyme secretion in neuronal cells [35,36]. Therefore, our experimental results revealed that VRSE treatment dramatically improved the nuclear Nrf-2 translocation and antioxidant enzyme expressions, such as GCLC, NQO1, and HO-1, in H_2_O_2_-treated HT22 cells (Figure 6). Therefore, VRSE exerts neuroprotective effects by promoting the antioxidant enzyme expression through Nrf-2 activation in oxidative stress-induced neuronal cells.

The pathogenesis of various neurodegenerative diseases is closely related to the neuroinflammatory process, which can be triggered by various biological stimuli, such as glial reactions and oxidative stress [19]. Microglia are major resident immune cells and play an important role in mediating inflammatory responses in the CNS. Microglia-mediated neuroinflammation is considered a major source of ROS and reactive nitrogen species in the brain [37]. Excessively activated microglia by LPS stimulation can lead to neuronal cell death and generate pro-inflammatory factors, including TNF-α, IL-6, and IL-1β. Our study results revealed that VRSE significantly decreased the TNF-α and IL-6 secretion in LPS-activated BV2 microglial cells (Figure 4). Additionally, we evaluated the MAPK phosphorylation and NF-κB signaling pathway activation to investigate the potential mechanisms of anti-neuroinflammatory effects in LPS-stimulated BV2 cells. NF-κB, as a key transcription factor, plays an essential role in immune and inflammatory responses. Inactivated p65 of NF-κB is located in the cytoplasm in unstimulated cells bound to inhibit NF-κB alpha (IκBα). Microglial cell stimulation by inflammatory stimulants, such as LPS, significantly enhances the phosphorylation and degradation of IκBα, which then frees NF-κB to translocate into the nucleus to promote pro-inflammatory factors [37]. Hence, we decided to measure the p65 expression level in cytoplasmic and nuclear extracts. Western blot analysis revealed that p65 translocation into the nucleus strongly increased by LPS stimulation, whereas VRSE treatment notably inhibited nuclear NF-κB p65 migration in microglia BV2 cells. The MAPK pathway plays a significant role in the LPS-treated expression of several endogenous inflammatory factors, as well as NF-κB activation [38,39]. Therefore, we evaluated the effect of VRSE on LPS-stimulated phosphorylation of MAPK signaling and revealed that VRSE remarkably inhibited the phosphorylation of ERK, p38, and JNK in a concentration-dependent manner. These results demonstrated that the anti-neuroinflammatory effect of VRSE can inhibit several pro-inflammatory factors in the microglial BV2 cells by blocking NF-κB and MAPK signaling pathways. Therefore, the targeting inhibition of the overproduction of inflammatory factors is a valuable clinical approach for neurodegenerative disease treatment.

We performed phytochemical analyses using HPLC to investigate the relationships between the VRSE physiological activities and its components. Our result identified vitexin and isovitexin as the main VRSE component. Previous studies revealed that vitexin exerts neuroprotective effects against isoflurane-induced neurotoxicity by targeting the TRPV1 and NR2B signaling pathways in rats and PC12 cells [40]. Additionally, vitexin attenuates LPS-induced lung inflammation by activating the Nrf-2 pathway [41]. Further, isovitexin has neuroprotective activity against glutamate-induced cell damage in mice hippocampal slices [42]. Moreover, isovitexin exerts anti-inflammatory and antioxidant activities on LPS-induced lung injury by inhibiting MAPK and NF-κB and activating HO-1/Nrf-2 pathways [43]. Therefore, the neuroprotective and anti-neuroinflammatory effects of VRSE can reflect the presence of vitexin and isovitexin.

## 5. Conclusions

In conclusion, this study revealed that VRSE alleviated H_2_O_2_-induced cytotoxicity in HT22 cells via ROS accumulation and apoptosis inhibition. Additionally, VRSE exhibited neuroprotective effects through TrkB/CREB/BDNF pathway and Nrf-2/antioxidant enzyme activation. Further, we proved that VRSE ameliorated neuroinflammatory reactions in LPS-stimulated microglia BV2 cells through NF-κB and MAPK inhibitions. Moreover, the main components of VRSE, including vitexin and isovitexin, may be closely related to its neuroprotective and anti-neuroinflammatory effects. Therefore, VRSE may be a potential candidate for preventing and treating neurodegenerative diseases related to neurotoxicity, oxidative stress, and neuroinflammation.

## Figures and Tables

**Table 1:** Primary and secondary antibodies use for Western blot analysis.

Antibody	Corporation	Product No.	RRID	Dilution Rate
BAX	Cell Signaling	#2772	AB_10695870	1:1000
Bcl-2	Cell Signaling	#3498	AB_1903907	1:1000
AIF	Cell Signaling	#4642	AB_2224542	1:1000
C-CAS-3	Cell Signaling	#9664	AB_2070042	1:1000
CAS-3	Cell Signaling	#9662	AB_331439	1:1000
C-PARP	Cell Signaling	#9548	AB_2160592	1:1000
PARP	Cell Signaling	#9532	AB_659884	1:1000
β-actin	Cell Signaling	#4970	AB_2223172	1:1000
NF-κB p65	Cell Signaling	#8242	AB_10859369	1:1000
Lamin B1	Cell Signaling	#13435	AB_2737428	1:1000
P-ERK	Cell Signaling	#4377	AB_331775	1:1000
ERK	Cell Signaling	#9102	AB_330744	1:1000
P-p38	Cell Signaling	#9211	AB_331641	1:1000
P38	Cell Signaling	#9212	AB_330713	1:1000
P-JNK	Cell Signaling	#9251	AB_331659	1:1000
JNK	Cell Signaling	#9252	AB_2250373	1:1000
Nrf-2	Cell Signaling	#12721	AB_2715528	1:1000
HO-1	Cell Signaling	#82206	AB_2799989	1:1000
NQO1	Santa Cruz	#SC-32793	AB_628036	1:1000
GCLC	Thermo Fisher	#PA5-87854	AB_2804457	1:1000
P-TrkB	Cell Signaling	#4621	AB_916186	1:1000
TrkB	Cell Signaling	#4603	AB_2155125	1:1000
P-Akt	Cell Signaling	#4060	AB_2315049	1:1000
Akt	Cell Signaling	#4691	AB_915783	1:1000
P-CREB	Cell Signaling	#9191	AB_331606	1:1000
CREB	Cell Signaling	#9197	AB_331277	1:1000
BDNF	Cell Signaling	#47808	AB_2894709	1:1000
2nd anti-mouse	Cell Signaling	#7076	AB_330924	1:5000
2nd anti-rabbit	Cell Signaling	#7074	AB_2099233	1:5000

## Data Availability

The data are contained within the article.
